# Optimizing Growth Regulator Concentrations for *Cannabis sativa* L. Micropropagation

**DOI:** 10.3390/plants14162586

**Published:** 2025-08-20

**Authors:** Gabrielle A. Johnson, Carissa L. Jackson, Antonio Timoteo, Papaiah Sardaru, Michael H. Foland, Purushothaman Natarajan, Sadanand A. Dhekney

**Affiliations:** Department of Agriculture, Food and Resource Sciences, University of Maryland Eastern Shore, 1 College Backbone Road, Princess Anne, MD 21853, USAaajunior@umes.edu (A.T.J.); pnatarajan@umes.edu (P.N.)

**Keywords:** auxin, contamination, cytokinin, hyperhydricity, industrial hemp

## Abstract

In this study, the effect of growth regulators on shoot proliferation and rooting were evaluated to develop an efficient micropropagation protocol for the *Cannabis sativa* L. cultivars ‘Cherry Soda’ and ‘Purple’. Apical meristems were isolated from actively growing shoots of stock plants and transferred to Driver and Kuniyuki Walnut (DKW) culture medium containing either 0.0, 0.5, 1.0, 2.0, or 5.0 μM meta-Topolin to study their shoot proliferation response. Resulting shoot cultures were transferred to medium containing varying levels of Indole Acetic Acid (IAA), Indole Butyric Acid (IBA), or Naphthalene Acetic Acid (NAA), solely or in combination, and were subjected to a 10-day dark incubation followed by a 16 h/8 h light/dark period to identify the best treatment for root production. Among the different shoot proliferation treatments studied, the maximum number of shoots was produced on the control medium that was devoid of any meta-Topolin. Cultures grown on medium containing 5.0 μM meta-Topolin exhibited hyperhydricity, where shoots appeared translucent and pale green in color; were characterized by water-soaked lesions; and leaves appeared curled and brittle in contrast to healthy looking cultures. Among the various rooting treatments studied, shoots grown in the dark for 10 days exhibited the highest frequency of rooting on medium containing 4.0 μM NAA or 6.0 μM IBA + 1.0 μM NAA. Full developed plants with a robust shoot and root system were transferred to soil, acclimatized under conditions for high humidity, and then transferred to ambient conditions in 4 weeks. The micropropagation protocol developed here allows for rapid multiplication of disease-free plants in *C. sativa* cultivars.

## 1. Introduction

*Cannabis* is an annual, short day plant that is valued for its multipurpose uses in food, fiber, medicine and cosmetic products [1]. Botanists and taxonomists differ on whether the *Cannabis* genus is composed of three species *C. sativa*, *C. indica* and *C. ruderalis* or if these are subspecies within *Cannabis sativa* [2,3,4]. *Cannabis* cultivars may be classified as drug-type or fiber type [1] depending on the type and level of cannabinoids present in various plant types. Drug-type cultivars are typically characterized by high levels of delta 9 tetrahydrocannabinol (THC), which is responsible for the psychoactive effects and used for recreational or medicinal purposes. Fiber type cultivars on the other hand are characterized by high levels of cannabidiol (CBD) while containing trace levels of THC and other cannabinoids [5]. Cultivars can also be distinguished from their growth habits with drug-type cultivars exhibiting short and bushy growth [6], while fiber type cultivars produce single stems that are 6–8 feet in length. The 2018 U.S. farm bill legally classified any *Cannabis* cultivars that produce less than 0.3% total THC as industrial hemp [7] and have been utilized for seed (oil, food and feed), fiber and cannabinoid production.

*Cannabis sativa* has 10 pairs of chromosomes which include nine autosomes [6] and one pair of sex chromosomes that determines the sex of the plant [6]. *Cannabis* plants are predominantly dioecious where male and female flowers are borne on different plants [8]. Female flowers are characterized by the dense production of trichomes that are the site of accumulation of several secondary metabolites including cannabinoids, terpenes and phenolic compounds [9,10]. Male flowers in contrast have few trichomes with negligible production of secondary metabolites due to pollen sacs [1]. Thus, *Cannabis* cultivation for the production of and use of its medicinal compounds involves the exclusive use of female plants. Since seedling populations segregate into plants with male and female flowers, *Cannabis* plants for cannabinoid production are obtained by clonal propagation using cuttings or through feminized seed production [11]. Softwood cuttings obtained from rapidly growing shoots of growing female plants produce roots in 8–10 days.

Feminized seed production involves treating female hemp plants with plant growth regulators to induce sex reversal, leading to the development of male flowers and self-fertilization. Major disadvantages of vegetative propagation include the requirement of a large area under indoor conditions for maintaining stock plants and the potential transmission of pathogens from infected stock plants. *Cannabis* production is adversely affected by several viral pathogens and fungal pathogens, which can cause a significant decrease in the yield and quality of fiber, flower and seed. Viral pathogens are primarily transmitted via vegetative propagation when infected stock plants are used to obtain clonal plant material while other pathogens can spread through seed. Viral pathogens such as hop latent viroid and beet curly top virus have ravaged indoor *Cannabis* cultivation and threaten to impact field production of industrial hemp [12,13,14]. The production of disease-free, clean planting material is important for the profitability and sustainability of the *Cannabis* industry.

Large-scale propagation of uniform, disease-free female plants can be achieved through in vitro culture techniques such as micropropagation [1]. Micropropagation is a method of asexual propagation that involves the isolation of shoot apical meristems from stock plants and their culture on nutrient medium containing plant growth regulators for obtaining disease-free plants [1]. Shoot apical meristems are the desired choice of explant for deriving micropropagation cultures due to their undifferentiated state of development and lack of vascular organization, which ensures cellular freedom from endophytic pathogens including viruses and viroids [15,16]. *Cannabis* micropropagation is influenced by several factors including genotype, explant type and developmental stage, media nutrients, growth regulator concentration and combinations and environmental factors including photoperiod, light intensity and temperature [17,18]. In vitro culture of *Cannabis* has been previously reported from various cultivars and involves the use of explants including one–three nodes, stem, leaf and petiole segments, seedlings and female inflorescences [19,20,21,22]. However, there are no studies that describe the development of a *Cannabis* micropropagation protocol using shoot apical meristems. The use of shoot apical meristems as starting explant material highly desired for the production of disease-free, clean plant material [16]. *Cannabis* cultivars exhibit a wide variation in their plant regeneration response on different media treatments and previously published protocols [23,24] could not be replicated for other drug-type genotypes [25,26]. Thus, there is a need to study the response of various *Cannabis* species and cultivars under in vitro culture conditions to optimize efficient micropropagation protocols. In the current study, we utilized shoot apical meristems to study the shoot proliferation and rooting response of two *C. sativa* cultivars ‘Purple’ and ‘Cherry Soda’ on varying concentrations and combinations of growth regulators. We report on the development of a successful micropropagation protocol for the production of *C. sativa* plant materials for cannabinoid production.

## 2. Materials and Methods

### 2.1. Plant Material

*Cannabis sativa* L. cultivars ‘Cherry Soda’ and ‘Purple’ were used for micropropagation experiments. The two genotypes are legally classified as industrial hemp cultivars based on their total THC levels, which permits them to be used for research purposes under the 2018 U.S. Farm Bill. Rapidly growing shoots from field-grown, female plants were obtained just after flower emergence and asexually propagated to produce rooted cuttings. The rooted cuttings were grown in 1- gallon pots containing commercial potting mix (Promix BX M™, Quakertown, PA, USA) and fertilized with 20 g 15N:9P:12K slow-release fertilizer (Osmocote™, ICL Growing Solutions, Dublin, OH, USA). After 4 weeks, the plants were transferred to 5-gallon pots containing the same substrate as above and fertilized every 3 months. Additionally, liquid fertilizer (General Hydroponics, Santa Rosa, CA, USA) consisting of 2 (nitrogen): 1 (phosphorus): 6 (potassium) was added at the rate of 5 mL per gallon of water during each irrigation. Stock plants were maintained in a vegetative phase by growing them in an indoor facility under 2000 µmol/m^2^/s light intensity and 18 h light/6 h dark photoperiod at 24^ο^ C and 30% relative humidity. Insect pest management was achieved using Azamax^®^ (active ingredient 1.2% azadirachtin), Flying Skull^®^ (0.05% citric acid) and Grandevo^®^ (*Chromobacterium subtsugae* PRAA4-1 strain). These stock plants were used to obtain explants for use in micropropagation experiments.

### 2.2. Medium Preparation

Media treatments for shoot proliferation and rooting experiments consisted of DKW macroelements, microelements and vitamins [27], 30 g L^−1^ sucrose, 0.1 g L^−1^ myoinositol and 0.3 g L^−1^ potassium nitrate (hereby designated as DM medium). Meta-Topolin and auxins at varying concentrations were added to the shoot proliferation and rooting media treatments, respectively. The pH of the medium was adjusted to 5.7 using 1.0 M KOH, prior to autoclaving at 121 degrees Celsius for 20 min at 15 psi. The medium was cooled to 55 °C after autoclaving and then poured into 16 × 100 mm Petri dishes or 16 oz Deli cup polypropylene containers (Pro-Kal^®^, Lake Forest, IL, USA). The media treatments were stored for 3–4 days at 25 °C prior to use in shoot proliferation and rooting experiments.

### 2.3. Culture Initiation

**Figure 1:**
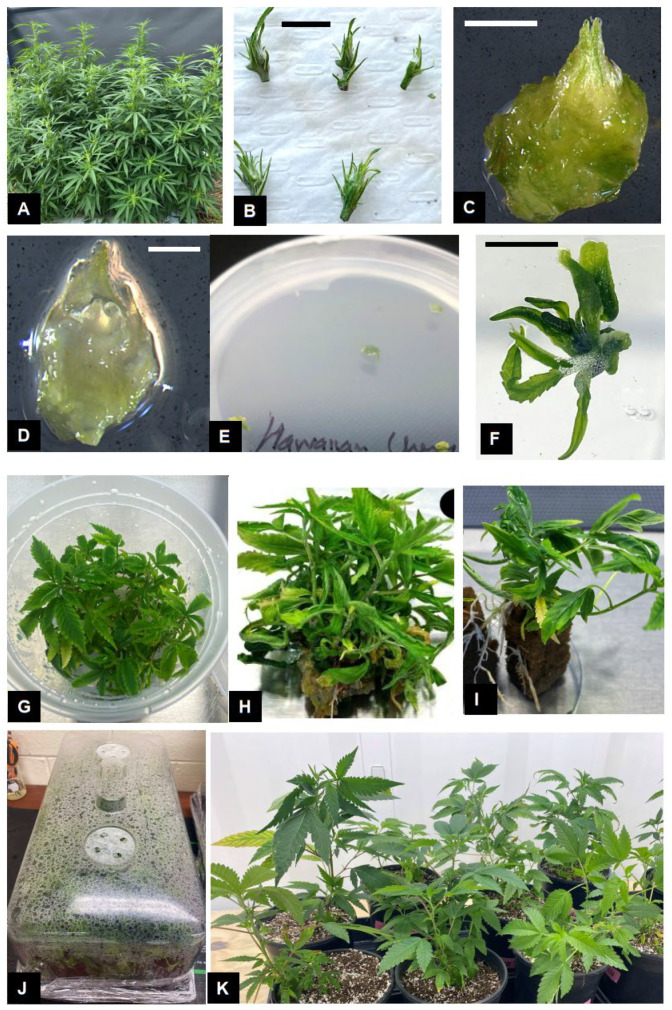
Micropropagation of *Cannabis sativa* L. Stock plants grown under indoor conditions (**A**) were used to obtain shoot tips (**B**) and excise the shoot apical meristem, comprising two leaf primordia (**C**) and the meristematic dome (**D**). Growth (**E**) and leaf production (**F**) from shoot apical meristem resulted in shoot proliferation (**G**,**H**). Rooting of shoots with 1–2 nodes (**I**) to produce fully developed plants. Fully developed plants are transferred to sterile potting mix and maintained under conditions of high humidity (**J**). Fully acclimated plants growing under ambient conditions (**K**). Scale bars: (**B**) = 10 mm, (**C**) = 1 mm, (**D**) = 0.2 mm, (**F**) = 5 mm.

Rapidly elongating shoots from 24-week-old stock plants (Figure 1A) were used to obtain explants for micropropagation cultures. Explants were prepared by excising 3–4 cm long shoot tips from the apical portion of shoots and the subtending leaves were removed under a dissecting microscope and discarded (Figure 1B). The explants were then rinsed in 70% ethyl alcohol for 30 sec and then washed in sterile distilled water. Explants were surface-sterilized by washing them in a 25% commercial bleach solution containing 1–2 drop of TWEEN 20^®^ for 5 min. Explants were then washed 3 times, 5 min each, with distilled water to remove the excessive bleach solution. Following surface-sterilization, unopened leaves from the explants (Figure 1C) were removed under a dissecting microscope to isolate the apical meristem (Figure 1D). The apical meristems (0.5–1.0 mm in length) were transferred to Petri dishes containing DM medium and maintained at 25 °C under a 16 h photoperiod of 120 μmol per m^2^/s light intensity from 20 W T8 LED Tube lights (Philips, Somerset, NJ, USA).

### 2.4. Influence of Meta-Topolin on Shoot Proliferation

After 4–5 weeks of meristem culture on DM medium, resulting shoots with at least two nodes were transferred to the Deli cup containers containing 200 mL of DM medium with either 0.0, 0.5, 1.0, 2.0, or 5.0 μM concentration of meta-topolin (mT). The cultures were maintained as described above. There were 5 shoots in each container with 5 replicate containers for each treatment. The number of shoots obtained from each Deli cup container were recorded prior to transfer to fresh medium at 4 weeks intervals. After 4 transfers, resulting shoots were transferred to media containing auxin treatments to study their rooting response.

### 2.5. Screening Cultures for Endophytic Contamination and Growth Abnormalities

In vitro cultures were routinely examined to record the presence of any endophytic contamination and distinguish it from contamination occurring due to improper worker techniques. Endophytic contamination was classified as any fungal and bacterial growth that seemed to originate from the cut end of meristematic explants or shoot tips following culture initiation and subsequent transfers. Fungal contamination was characterized by fuzzy growth while bacterial contamination was characterized by slimy growth. Any cultures exhibiting the presence of endophytes were immediately discarded. The proliferating shoot cultures were also observed for the presence of hyperhydricity, which was characterized as leaves and stems exhibiting a glassy or water-soaked appearance. Additionally, cultures were carefully examined for any in vitro flowering during the shoot proliferation phase. Any male or female flowers observed on shoot cultures were recorded, and such cultures were discarded during the transfer process.

### 2.6. Influence of Plant Growth Regulators and Photoperiod on Root Production

The influence of various auxin concentrations and combinations, and photoperiod on rooting of *C. sativa* ‘Purple’ shoots in liquid medium was studied. Three growth regulators, Indole acetic acid (IAA), indole butyric acid (IBA) and naphthalene acetic acid (NAA) were tested at different concentrations and combinations. Liquid DM medium was prepared as described above without the addition of TC agar. The growth regulator treatments were added to the liquid medium prior to autoclaving except for IAA that was filter sterilized using a 0.2-micron filter (Fisher Scientific, Waltham, MA, USA) that was attached to a 10 mL syringe (BD Luer-Lok™, Sigma-Aldrich, St. Louis, MO, USA). IAA was added to the liquid media treatments after cooling the medium to 55 °C. Four Oasis root cubes (1-inch, Oasis growers solutions, Kent, OH, USA) were transferred to each Deli cup container. The containers were then autoclaved at 121 °C and 15 psi. for 20 min. After cooling, 100 mL of liquid medium treatment was transferred to each Deli cup container to completely soak the root cubes. The control consisted of liquid DM medium with no auxin. Fully developed shoots, 2 inches in length, were inserted in each root cube and the containers were capped and sealed using plastic wrap. The containers were either directly maintained under light at 25 °C and 16 h photoperiod or transferred to the dark and maintained at the same temperature for 10 days. After 10 days, the containers were removed and transferred to light as described above. There was a total of three replicate containers for each treatment and the control. The number of roots producing shoots in each treatment and the control was recorded after 5 weeks of growth in the liquid medium.

### 2.7. Plant Acclimatization

Acclimatization was carried out in plastic trays containing 50 celled plug inserts and a plastic dome that was used to maintain relative humidity. Plants growing in root cubes were transferred to the inserts covered with sterile potting mix. Plants were sprayed with distilled water to maintain 100% relative humidity. Trays were then covered with a plastic dome and sealed with plastic wrap to ensure transferred plants did not desiccate from moisture loss. High relative humidity was maintained for 10 days by spraying domes with distilled water. After 10 days, the dome vents were open to gradually decrease the humidity. The domes were completely removed after 3 weeks and plants were maintained under indoor conditions for 1 week. Fully acclimated plants were then transferred to quart-sized plastic pots containing commercial potting mix (Promix BX M™, Quakertown, PA, USA) and transferred to and indoor growth facility.

### 2.8. Statistical Analyses

A completely randomized design was used for this experiment. The data was analyzed using one-way analysis of variance (ANOVA) while treatment means were compared using post hoc analysis through Tukey test for contrasts at a confidence level of 95% (*p* ≤ 0.05). Data analysis was performed using RStudio (version 2024.12.1) with the Rcmdr package (version 2.9-5) in R (version 4.4.3). A two-way ANOVA was conducted to evaluate the effects of cultivar, meta-Topolin concentration (treatment), and their interaction on shoot proliferation. A regression analysis was conducted to examine the relationship between meta-Topolin concentration and shoot proliferation in ‘Purple’ and ‘Cherry Soda’.

## 3. Results and Discussion

### 3.1. Effect of mT Concentration on Shoot Proliferation

Shoot apical meristems excised from shoot tips explants developed visible leaf structures after 3 weeks of growth on the culture medium (Figure 1E,F). Rapid growth and shoot proliferation was observed during the subsequent transfers to fresh media treatments (Figure 1G,H).

**Figure 2:**
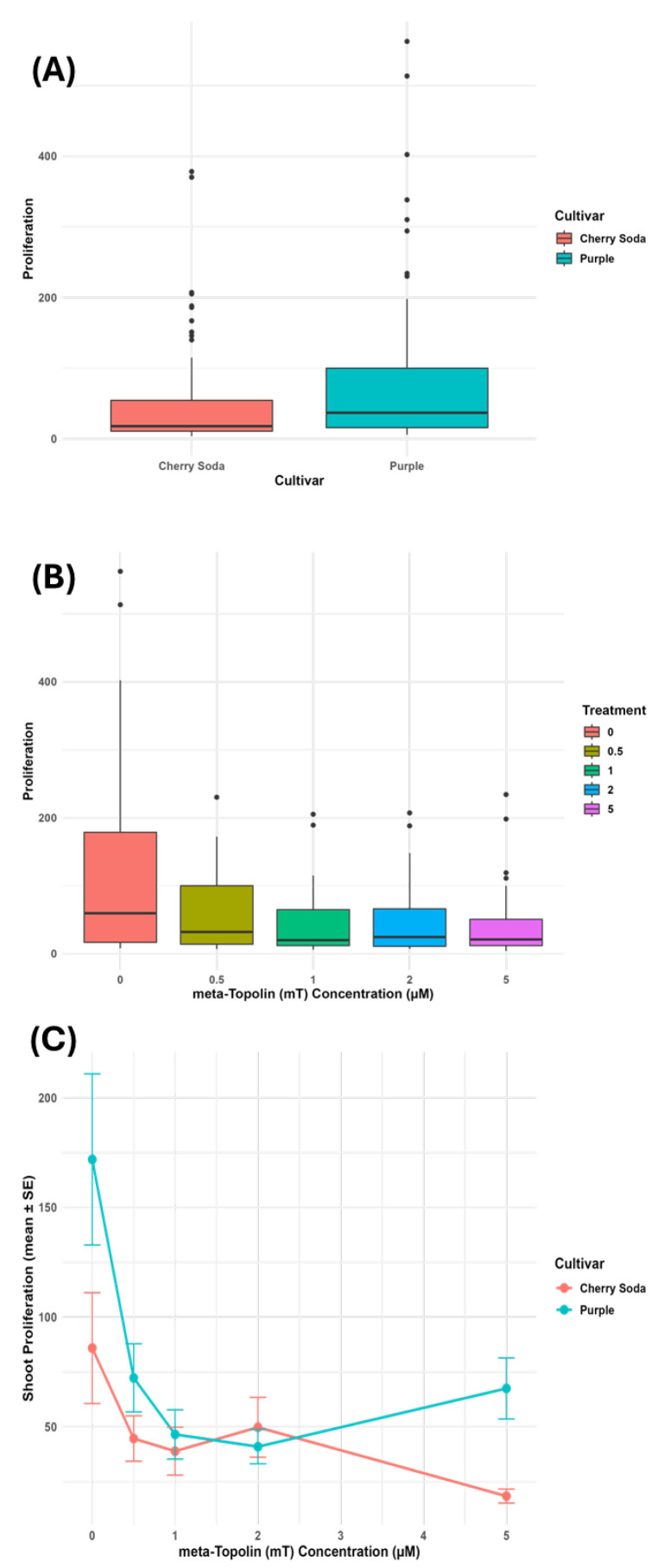
Boxplots showing shoot proliferation across cultivars (**A**), treatments (**B**), and the cultivar × treatment interaction (**C**). Proliferation represents the mean number of shoots per explant pooled across all four subculture periods. Values represent means and standard error (SE) from five replications per treatment. Statistical analysis was performed using two-way ANOVA to assess the main and interaction effects of cultivar and mT concentration.

The effect of four meta-topolin (mT) concentrations on the number of shoots produced in *Cannabis sativa* ‘Purple’ and ‘Cherry Soda’ was studied over a period of 16–20 weeks. After 4 weeks of culture initiation, the highest number of shoots were observed in ‘Purple’ on the control medium (14.4) followed by shoot cultures growing on DM medium with 5.0 μM mT (13.60), 0.5 μM mT (13.00), 1.0 μM mT (11.00), and 2.0 mT (9.60). At this stage, there were no significant differences in the number of shoots produced on the control and different growth regulator treatments (Table 1). However, starting with the second subculture that occurred approximately 8–10 weeks after culture initiation, shoot cultures growing on the control medium produced the highest number of shoots (54.80), followed by medium with 5.0 μM mT (29.60), 0.5 μM mT (28.40), 1.0 μM mT (25.00), and 2.0 μM mT (23.80). While the control medium produced a significantly higher number of shoots, there was no difference between shoot cultures grown on other concentrations of mT. A similar trend was observed during the third and fourth subculture. At the end of the fourth subculture that occurred 16–20 weeks after culture initiation, the highest number of shoots were observed in the control medium (370.2) followed by 0.5 μM mT (174.5), 5.0 μM mT (130.00), 1.0 μM mT (102.67), and 2.0 μM mT (85.40). Overall, the control produced the highest number of shoots (Table 1). A similar trend was observed in ‘Cherry Soda’ where there was no significant difference in shoot proliferation rates among different treatments during the first transfer (Table 2). However, the control exhibited the highest shoot proliferation rate (210.6) during subsequent transfers followed by cultures grown on medium containing 2.0 μM mT (124.0) and 0.5 μM mT (110.4). Among the two cultivars tested, ‘Purple’ had a higher shoot proliferation rate (370.2) and potential for plant production compared to ‘Cherry Soda’ (210.6). The micropropagation response of *C. sativa* cultivars is highly influenced by genotype, explant type and developmental stage, growth regulator concentration and combination and culture conditions [26]. Our results are similar to previous results obtained for *Cannabis* shoot multiplication where cultures were grown in the absence of any growth regulators and were multiplied using a hedging technique, that consisted of excising growing shoots at 3-week intervals [18]. In other studies, no significant differences were observed in nodal segment-derived *C. sativa* cultures that were grown either on medium containing various cytokinins or the control medium without any growth regulators [25]. However, the nodal position of the explant on the donor plant influenced the rate of shoot proliferation with nodes closer to the apical meristem producing fewer axillary shoots than the distal ones. Similarly, efficient shoot production and rooting was observed without any growth regulators in the culture medium when hypocotyl explants from germinated seedlings were used [21]. In other studies, efficient shoot proliferation was observed on medium containing either MS salts [28] or DKW salts [27] containing various cytokinins including thidiazuron (TDZ), mT, benzyl amino purine (BAP), N6-(2-isopentenyl) adenine (2iP) and kinetin or gibberellins and auxins [17,24,29,30,31,32]. In these studies, different *C. sativa* genotypes and explants were used to establish cultures. A two-way ANOVA was performed on pooled data across all four subculture periods to evaluate the effects of cultivar, meta-topolin (mT) concentration (treatment), and their interaction on shoot proliferation. Here, proliferation refers to the mean number of shoots per explant aggregated across subcultures. The analysis revealed a significant main effect of cultivar (F_1,186_ = 8.16, *p* = 0.0048), with ‘Purple’ producing significantly more shoots than ‘Cherry Soda’. A highly significant main effect of treatment was also observed (F_4,186_ = 8.40, *p* < 0.001), indicating that mT concentration strongly influenced shoot proliferation. The interaction between cultivar and treatment was not statistically significant (F_4,186_ = 2.11, *p* = 0.081), suggesting both cultivars responded similarly to varying mT levels. Post hoc Tukey’s HSD analysis confirmed that shoot proliferation on the control medium (0 µM mT) was significantly greater than at all other concentrations (0.5–5 µM) across both cultivars. These results are visualized in Figure 2A–C, and the ANOVA summary (Table 3).

**Figure 3:**
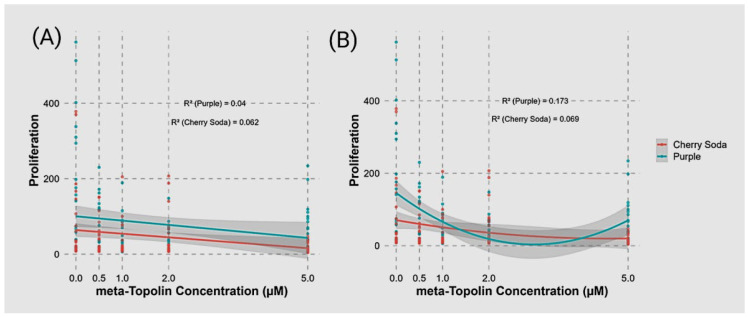
Linear regression of shoot proliferation for ‘Purple’ (blue) and ‘Cherry Soda’ (red) across meta-Topolin concentrations (**A**). Linear regression lines with confidence intervals are shown for each cultivar. The R^2^ values indicate the proportion of variance explained by the linear regression: R^2^ = 0.04 for ‘Purple’ and R^2^ = 0.062 for ‘Cherry Soda’. (**B**) Polynomial regression (degree 2) of shoot proliferation across meta-Topolin concentrations for the same cultivars. Polynomial regression captures the non-linear relationship between meta-Topolin concentration and proliferation, with R^2^ = 0.173 for ‘Purple’ and R^2^ = 0.069 for ‘Cherry Soda’. Shaded regions represent 95% confidence intervals for the regression lines.

Similarly, regression analyses revealed a negative relationship between meta-Topolin concentration and shoot proliferation, with both cultivars showing a decline in proliferation as meta-Topolin levels increased (Figure 3A). The R^2^ values for the linear regression model were 0.04 for ‘Purple’ and 0.062 for ‘Cherry Soda’, indicating that meta-Topolin concentration explained only a small proportion of the variability in shoot proliferation for both cultivars. To better capture potential non-linear trends, a polynomial regression model (degree 2) was fitted for each cultivar (Figure 3B). The polynomial regression model improved the fit for ‘Purple’, with an R^2^ of 0.173, suggesting a stronger relationship between meta-Topolin concentration and shoot proliferation compared to the linear model. For ‘Cherry Soda’, the polynomial model slightly improved with an R^2^ of 0.069. Both models indicated that the most pronounced reduction in proliferation occurred at lower meta-Topolin concentrations (0–1 µM), with a more gradual decline or stabilization at higher concentrations (2–5 µM). *Cannabis* species can be classified into seed/fiber- and drug-type cultivars based on their use for commercial production. The fiber/seed-type cultivars are characterized by the production of a single long stem without any branching, while drug-type cultivars exhibit short bushy growth with several branches [33]. Such morphological and growth differences between cultivars may be manifested under in vitro conditions and the response to shoot proliferation on various media treatments. A difference in the levels of endogenous plant hormones in explants of various genotypes may also contribute to a variable shoot proliferation response. An analysis of *C. sativa* plant tissues for endogenous hormones revealed high levels of cytokinins, especially trans zeatin, in the shoot apex and meristematic tissues [34]. The best shoot proliferation response may ultimately be observed from the interaction of cultivar, endogenous hormones in explants and the medium composition, which controls the expression of genes responsible for organogenesis and plant regeneration. These contrasting results highlight the importance of the influence of the genotype on shoot proliferation response in different media combinations. In the current study, the effect of mT on shoot proliferation was examined. Although various cytokinins are routinely used for micropropagation, some exhibit negative effects on plant regeneration due to their high stability in the culture medium and conversion into toxic metabolites over time [35,36]. Such limitations can be overcome by the use of cytokinins such as mT, which is less stable and may exhibit significantly lower carryover effects during rooting and plant acclimatization [37]. Based on these observations, mT was selected as the choice of cytokinin to study shoot proliferation.

### 3.2. Endophytic Contamination and Growth Abnormalities in Shoot Cultures

**Figure 4:**
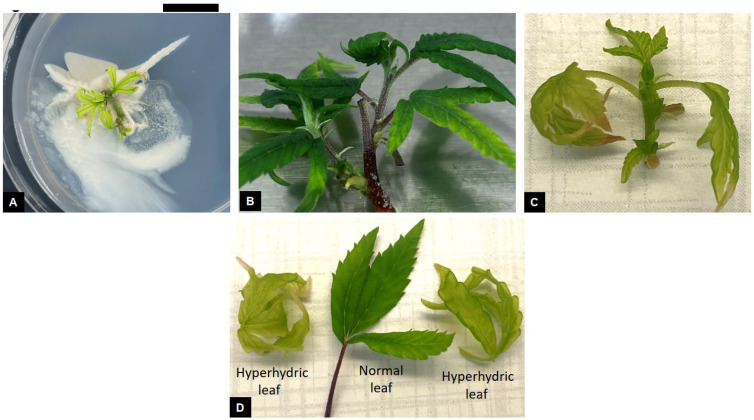
Endophytic contamination and growth abnormalities in *Cannabis sativa* L. Bacteria contamination can be observed occurring from the cut end of the explant (**A**). Occurrence of hyperhydricity in shoot cultures where normal shoots appear dark green in color (**B**) compared to hyperhydric shoots characterized by translucent appearance (**C**) with chlorotic, curled leaves (**D**). Scale bar: A = 5 mm.

Sporadic endophytic contamination was observed in a few cultures following initiation and shoot proliferation. Bacterial endophytes, characterized by white slimy growth was observed from explants on a single Petri dish (Figure 4A). Such explants were immediately identified and discarded. Similar endophytic contamination was observed in shoot tip explants and cultures of *C. sativa* ‘TJ’ that were established from greenhouse-grown plants [32]. Microbial contamination in micropropagation cultures is commonly observed in several plant species [38]. Endophytes can negatively influence in vitro cultures by slowing growth rate of cultures, causing inconsistent shoot and root production, and in severe cases, result in total culture loss [39,40]. Some endophytes may also be pathogenic and have the potential to produce plant disease [41]. A diverse range of microbial bacterial and fungal endophytes have been observed in *C. sativa* [42]. Endophytic contamination in cultures is observed after a few weeks of culture establishment but in certain instances, endophytes can stay latent for several years in apparently clean cultures before being manifested in the growth medium [39]. In previous *Cannabis* micropropagation studies, explants were found to exhibit extremely high rates of endophytic contamination despite being subjected to rigorous surface-sterilization techniques [17,32]. This was attributed to the explant type and source plants from which they were obtained. The use of shoot apical meristems explants for initiating *Cannabis* cultures resulted in a significantly lower level of contamination compared to the use of nodal explants [17]. In our studies, all cultures were established after carefully excising the shoot apical meristems under a dissecting microscope (Figure 1C,D). This might be the reason for the low level of endophytic contamination observed in cultures and a high shoot proliferation rate. Meristem tip culture is the most widely used method for large-scale production of clean, disease-free plant materials. Micropropagation using meristem tip culture involve the careful excision of the shoot apical meristem under a dissecting stereomicroscope [43]. Such explants consist of the apical meristem along with a few leaf primordia and are typically between 0.5 and 1.0 mm in size. Apical meristems are the preferred choice of explants for elimination of endophytes due to the undifferentiated state of cells in the meristematic dome, which lack vascular differentiation and are rapidly dividing, which may result in exclusion of microbes from this region [16,43]. Successful excision of shoot apical meristems and their subsequent culture can result in rapid shoot proliferation and production of healthy, disease-free clonal plant material. Although micropropagation largely preserves clonal fidelity of regenerated plants, somaclonal variations may be introduced during various stages of culture [44] and are considered undesirable from a commercial point of view. The occurrence and extent of somaclonal variation is dependent on the explant type and developmental stage, culture duration, plant regeneration pathway and medium composition [45]. Genetic analysis of regenerated plants can assist in the identification of any somaclonal variations occurring during the culture process [24,25]. A large number of somaclonal variants were observed in micropropagation-derived *Cannabis* plants following prolonged maintenance under in vitro conditions [46]. Genetic analysis of regenerated plants using genotype by sequencing (GBS) revealed a high frequency of genetic variation that could not be detected/identified by utilizing single-sequence repeat (SSR) markers. A large number of identified variations did not exhibit any visible phenotypic differences compared to the mother plants.

Among the two cultivars studied, ‘Purple’ produced a greater number of healthy shoots that exhibited normal shoot and leaf morphology (Figure 4B). Similarly, cultures exhibiting hyperhydricity were observed in cultures with higher mT concentrations (Figure 4C). Hyperhydric cultures appeared translucent and pale green in color, were characterized by water-soaked lesions and leaves appeared curled and brittle in contrast to healthy looking cultures (Figure 4C,D). Hyperhydricity can occur from an abnormal ratio of nitrate: ammonium ions in the medium, excessive levels of cytokinins or the production of ethylene during culture growth. High cytokinin concentration in the culture medium may result in hyperhydricity [47,48]. Ivanova and Van Staden [49] found cytokinins (TDZ, BA, and zeatin) to increase hyperhydricity in *Aloe polyphylla* in comparison to media without synthetic phytohormones. It was also found that an increase in the level of cytokinins (BA and zeatin) promoted hyperhydricity as well [49]. Liu et al. [50] found cytokinins to increase hyperhydricity in *Allium sativum* L. using BA and kinetin. Wróbel et al. [51] stated that the use of TDZ on industrial hemp shoots tip and nodal segments caused morphological issues such as hyperhydricity, leaf narrowing and rolling, and decreased shoot growth. Page et al. [26] performed a preliminary experiment with MS media and 0.5 μM of TDZ and observed several negative effects such as hyperhydricity, decreases in shoot multiplication, and callusing. In preliminary studies with medium optimization, we observed similar effects from the use of MS macroelements and microelements, and TDZ in the culture medium. In the current study, mT at higher concentrations may have inhibited plant growth by increasing hyperhydricity, which resulted in reduced shoot proliferation [47,48,50]. This may explain why more shoots were produced on control media. *Cannabis* is a fast-growing plant [52], which might be indicative of a high level of phytohormones that promote rapid growth and development. Thus, the addition of exogenous cytokinins may have caused an imbalance of the total levels in cultures, leading to hyperhydricity and poor shoot proliferation rate. Therefore, phytohormones may not be required for shoot proliferation in certain cultivars. Although, it is important to note that each cultivar reacts differently in vitro, which indicates the need for cultivar specific protocols to be developed when optimizing micropropagation protocols.

Cytokinins in the culture medium may influence ethylene biosynthesis [48,53]. Žd’árská et al. [48] found cytokinins to upregulate ethylene biosynthesis in *Arabidopsis* during root growth. In tissue culture, ethylene accumulates in culture vessels due to plant wounding, typically during transfer to fresh medium or as a stress response [1]. Ethylene production during the shoot proliferation stage can negatively influence culture growth [54]. Ethylene accumulation at high levels promotes hyperhydricity [55]. Hyperhydricity can cause a significant reduction in culture growth including shoot proliferation, poor rooting, and a low plant regeneration rate in tissue culture. Lack of chlorophyll is also a symptom of hyperhydricity [56]. A major cause of hyperhydricity is when the apoplast, which is the intercellular spaces within plants, is flooded with water instead of gas [56]. Van Den Dries et al. [56] found that air volume in the apoplast of hyperhydric *Arabidopsis* seedlings decreased significantly while water volume increased. When water in the culture vessel increases, due to high humidity or type of gelling agent, the apoplast can flood, resulting in hyperhydricity [56]. It was reported that an enzyme involved in ethylene biosynthesis is elevated when explants are hyperhydric [56]. High levels of auxin biosynthesis during rapid cell division and expansion also leads to excess water uptake and shoot deformation, resulting from hyperhydricity [54]. Culture hyperhydricity can be alleviated by increasing agar concentrations, using vented culture vessels to reduce humidity or release ethylene, altering the nitrate: ammonium ratio and/or decreasing the amount of cytokinin in culture media [30,57].

**Figure 5:**
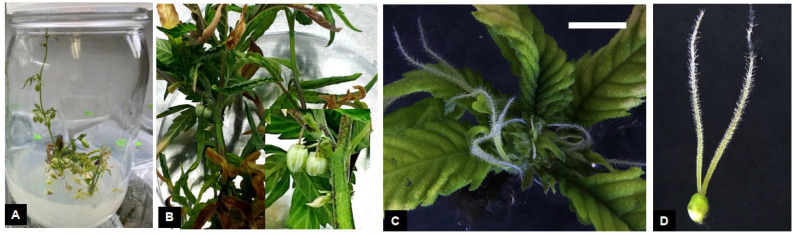
*In vitro* flowering in *Cannabis sativa* L. Occurrence of male (**A**,**B**) and female inflorescences (**C**) in proliferating shoot cultures. Female flower parts including the stigma, style and ovary (**D**). Scale bars: (**C**) = 10 mm, (**D**) = 1 mm.

The incidence of in vitro flowering during culture proliferation was also observed. Male and female inflorescences were observed at various stages of culture growth and development (Figure 5). Normal floral morphology was observed in both male and female flowers, which was characterized by the presence of stamens and pistils (Figure 5B,D). Such cultures exhibited slow growth and eventual growth termination. *Cannabis* is a dioecious plant species where male and female flowers are borne on separate plants. In our studies, cultures were initiated and established from female stock plants with confirmed sex under field conditions. The production of male flowers in such cultures may be indicative of a stress response encountered during in vitro culture [58]. Since *Cannabis* is an annual species, in vitro flowering during micropropagation is undesirable, may result in a decline in shoot proliferation and eventually termination of cultures [59]. Ethylene biosynthesis and accumulation in culture vessels may also lead to in vitro flowering [1]. The use of culture vessels with vented caps may lead to a decrease in hyperhydricity, ethylene accumulation and significantly improve the quality of cultures and the response to micropropagation [18,30]. Alternatively, addition of chemicals such as silver nitrate and polyamines including spermidine and spermine to the culture medium can decrease the biosynthesis of ethylene and its subsequent negative effects such as in vitro flowering and hyperhydricity [47,60].

### 3.3. Effect of Auxin Concentration and Combination on Rooting

The effect of different auxin concentrations and combinations on rooting of *C. sativa* ‘Purple’ shoots was tested under dark and light photoperiod environments. Among the various combinations tested (Table 4), the maximum rooting (66.67%) was observed in shoots cultured in the dark on DKW medium containing either 4.0 μM NAA or 6.0 μM IBA plus 1.0 μM NAA (Table 4). This was followed by shoots cultured on DKW medium containing 2.0 and 4.0 μM NAA under light conditions (53.3%). Rooting was profuse and fine root hair were observed in regenerated plants (Figure 1I). Thus, a combination of auxins along with a dark photoperiod treatment resulted in the best rooting percentage and plant regeneration. These results are similar to previous studies where IBA and NAA alone or in combination exhibited successful in vitro rooting in *Cannabis* [61,62]. Rooting of *C. sativa* shoots has been obtained on different media and growth regulator combinations [63]. Mostly commonly used auxins for rooting include IBA and NAA [23,24]. In some cases, 2,4-D was included in the medium to induce rooting [17], while efficient rooting was observed without any growth regulators in other studies [64]. Root morphology may be influenced by auxin type and concentration, with callus production being observed during the rooting process [32]. We did not observe any callus formation or abnormalities during the rooting process. This might be attributed to the genotypic response and the use of Oasis root cubes. A similar response was observed when substrates such as Oasis cubes and spaghnum peat moss-derived sponge were along with high IBA concentrations were used for rooting of *Cannabis* shoots [18,62]. Such substrates are more porous and may provide better conditions for root development compared to solid agar. Among the two photoperiod treatments, higher rooting was observed in the dark treatment. Similar results were observed in several genotypes of *Prunus avium* where a combination of IBA plus a 4–7-day dark treatment significantly improved in vitro rooting of shoot cultures [65]. Other studies that utilized a dark treatment demonstrated similar benefits of improved rooting [66,67]. Several morphogenic processes are controlled by auxin biosynthesis and its interaction with light [68]. Imposing a dark treatment may have resulted in a decrease in light-induced degradation of auxins in the culture medium, altered polar auxin transport within the plant cells and ultimately provided a favorable auxin balance for improved rooting [68]. Shoot etiolation was observed in the dark treatment but plants resumed normal growth and development following transfer to light.

### 3.4. Plant Acclimatization

Fully rooted shoots were transferred to sterile potting mix and maintained under conditions of high humidity (Figure 1J). The humidity was gradually reduced in both treatments and plants were completely acclimated in 4–5 weeks. Plants exhibited rapid growth when transferred to larger pots and maintained under 16 h light photoperiod. Greater than 95% of the plants were successfully acclimated and exhibited normal growth following transfer to ambient conditions (Figure 1K). Plant acclimatization is a critical phase of the micropropagation process due to drastic changes in environmental conditions encountered by regenerated plants. The development of stomata and subsequent gas exchange often slow when micropropagation-derived plants are transferred ex vitro [69]. This necessitates careful regulation of relative humidity to enable efficient plant acclimation from in vitro to ex vitro conditions.

## 4. Conclusions

**Figure 6:**
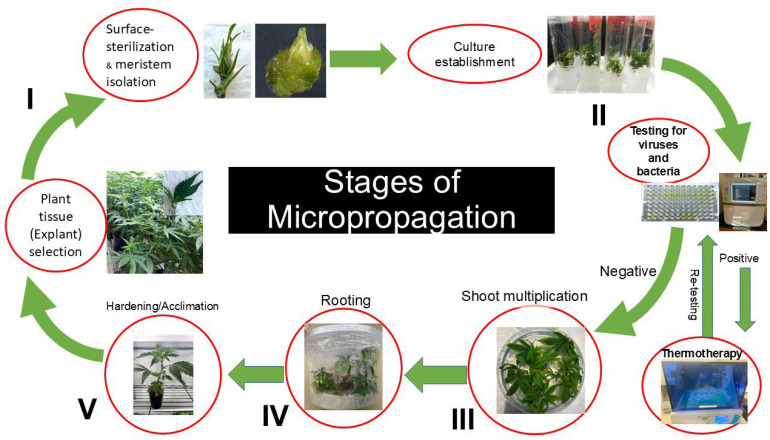
Flowchart for micropropagation of *Cannabis sativa* L. Phase I includes collection of shoot tips from actively growing female stock plants, their surface sterilization and isolation of apical meristems under a dissecting microscope. Phase II includes culture establishment and testing for the presence of endophytic pathogens, and their elimination. Phase III includes shoot proliferation for bulking up clean plant materials. Multiplied shoots are then rooted to produce fully developed plants in Phase IV, which are then acclimated under conditions of high humidity in Phase V and ready for commercial cultivation.

The findings of this study demonstrate that the *Cannabis sativa* cultivars ‘Purple’ and ‘Cherry Soda’ were able to produce a high number of shoots using DKW media without phytohormones. Rooting was also successful by including a dark treatment and the use of NAA and IBA. Several viral pathogens pose a serious threat to *Cannabis* cultivation with adverse effects on floral yield and quality. Most viral pathogens are transmitted through infected propagation materials including seed and softwood cuttings. Micropropagation of *C. sativa* is an important tool for the large-scale production of healthy, disease-free clonal plant materials. Since the micropropagation response is heavily influenced by genotype, it may be necessary to customize regeneration protocols for specific cultivars. Additional studies need to be conducted to test for plant pathogens during micropropagation to ensure the production of clean plant materials. Similarly, analyzing the genetic fidelity of micropropagation-derived plants and studying their field performance will help underscore the importance of using micropropagation for *Cannabis* propagation. We thus propose a protocol that depicts the various phases of micropropagation for the production of clean plant material (Figure 6). We envision that this protocol can be successfully used for commercial propagation of diverse *C. sativa* cultivars and ensuring the supply of healthy and disease-free clonal plant material.

## Figures and Tables

**Table 1:** Influence of meta-Topolin levels on shoot proliferation in *Cannabis sativa* ‘Purple’.

Meta-Topolin Level (μM)	Transfer Period			
	First	Second	Third	Fourth
0.5	13.00 ^a^	28.40 ^b^	93.60 ^b^	174.50 ^ab^
1.0	11.00 ^a^	25.00 ^b^	69.80 ^b^	102.67 ^b^
2.0	9.60 ^a^	23.80 ^b^	44.80 ^b^	85.40 ^b^
5.0	13.60 ^a^	29.60 ^b^	96.60 ^b^	130.00 ^b^
0	14.40 ^a^	54.80 ^a^	248.00 ^a^	370.20 ^a^

Five shoot tips were placed on each media treatment during every transfer, and resulting cultures were subcultured at 4–5-week intervals. The number of shoots produced per explant was recorded at each transfer period. Values represent means from five replications. Statistical analysis was performed using one-way ANOVA for each subculture period within each cultivar, followed by Tukey’s HSD test at a 95% confidence level (*p* ≤ 0.05). Means within each column followed by the same letter are not significantly different.

**Table 2:** Influence of meta-Topolin levels on shoot proliferation in *C. sativa* ‘Cherry Soda’.

Meta-Topolin Level (μM)	Transfer Period			
	First	Second	Third	Fourth
0.5	11.20 a	13.40 b	43.40 b	110.40 b
1.0	11.80 a	12.20 b	45.00 b	86.40 c
2.0	11.60 a	17.00 a	46.40 b	124.00 b
5.0	9.20 b	8.20 c	21.00 c	39.25 d
0	14.00 a	22.00 a	96.80 a	210.60 a

Five shoot tips were placed on each media treatment during every transfer, and resulting cultures were transferred at 4–5-week intervals. The number of shoots produced per explant was recorded at each transfer period. Statistical analysis was performed using one-way ANOVA for each subculture period within each cultivar, followed by Tukey’s HSD test at a 95% confidence level (*p* ≤ 0.05). Means within each column followed by the same letter are not significantly different.

**Table 3:** Two-way ANOVA results for the effects of cultivar, meta-topolin (mT) concentration (treatment), and their interaction on shoot proliferation.

Source	Df	Sum Sq	Mean Sq	F Value	Pr (>F)	Significance
Cultivar	1	52,643	52,643	8.163	0.00476	**
Treatment	4	216,582	54,145	8.396	3.01 × 10^−6^	***
Cultivar × Treatment	4	54,478	13,619	2.112	0.08100	.
Residuals	186	1,199,450	6449			

Significance codes: ***: *p* < 0.001; **: *p* < 0.01; .: *p* < 0.1.

**Table 4:** Influence of auxin concentration, combination and photoperiod on rooting of *Cannabis sativa* ‘Purple’.

Treatment	Rooting Percentage (%)	
	Light	Dark
IAA 4.0 μM	40.00 b	13.33 b
IAA 6.0 μM	33.33 b	20.00 b
IBA 4.0 μM	33.33 b	20.00 b
IBA 6.0 μM	46.67 b	20.00 b
IBA 6.0 μM + IAA 1.0 μM	26.67 b	13.33 b
IBA 6.0 μM + NAA1.0 μM	46.67 b	66.67 a
NAA 2.0 μM	53.33 a	20.00 b
NAA 4.0 μM	53.33 a	66.67 a
Control	26.67 b	20.00 b

Four shoots were transferred to Deli cup containers containing Oasis© cubes that were soaked in liquid media treatments. Containers were either placed under light at 25 °C and 16 h photoperiod or transferred to the dark and maintained at the same temperature for 10 days, prior to transfer to light. Values represent mean and standard error (SE) from 3 replications for each treatment. Statistical analysis was carried out using one-way ANOVA. Means within columns followed by the same letter were not significantly different according to the Tukey test at a confidence level of 95% (*p* ≤ 0.05).

## Data Availability

The original contributions presented in this study are included in the article. Further inquiries can be directed to the corresponding author.
